# Assessment of Irrigation Water Use Efficiency in Citrus Orchards Using AHP

**DOI:** 10.3390/ijerph18115667

**Published:** 2021-05-25

**Authors:** Rocío Poveda-Bautista, Bernat Roig-Merino, Herminia Puerto, Juan Buitrago-Vera

**Affiliations:** 1 INGENIO (CSIC-UPV), Institute of Innovation and Knowledge Management, Universitat Politècnica de València, 46022 Valencia, Spain; 2 Instituto de Investigación Para la Gestión Integrada de Zonas Costeras, Universitat Politècnica de València, 46730 Grao de Gandia, Spain; bernat@upv.es; 3 Centro de Investigación e Innovación Agroalimentaria y Agroambiental (CIAGRO-UMH), Miguel Hernández University, 03312 Orihuela, Spain; hpuerto@umh.es; 4 Department of Economics and Social Sciences, Universitat Politècnica de València, 46022 Valencia, Spain; jmbuitra@esp.upv.es

**Keywords:** water management, efficiency, citrus productivity, irrigation management, performance indicators, AHP, multicriteria decision analysis (MCDA)

## Abstract

Irrigation water use efficiency, the small size of the orchards, and part-time farmers are major issues for Spanish citriculture. How should irrigation water use efficiency be assessed? Does irrigation water use efficiency improve when increasing the size of the orchards? Are full-time farmers more efficient in irrigation water use than part-time ones? To address these three questions, we propose to apply a new multicriteria approach based on the analytic hierarchy process (AHP) technique and the participation of a group of experts. A new synthetic irrigation efficiency index (IEI) was proposed and tested using data from an irrigation community (IC) and a cooperative of farmers in the East of Spain. The results showed that the size of the orchards had no relation with the IEI scoring but full-time farmers tended to have better IEI scores and, thus, were more efficient. These results were obtained from a sample of 24 orchards of oranges, navelina variety, growing in a very similar environment, and agronomical characteristics. The proposed methodology can be a useful benchmarking tool for improving the irrigation water management in other ICs taking into account the issues related to farm data sharing recorded during the case study.

## 1. Introduction

Constrained by a Mediterranean climate, irrigation is essential for citrus production in Spain. According to FAOSTAT data of 2019 [1], Spain is the largest producer of oranges and mandarines in the EU-27, as it accounts for 57% of the cropped area and 56% of the production. The Valencian community is the main producer of oranges and mandarines among the Spanish regions with 49% and 67% of the oranges and mandarines cultivated areas in Spain, respectively [2]. Despite the long agricultural tradition of the Valencian community, in recent decades there has been a tendency to abandon the land, even on fertile irrigated land. The lack of generational replacement and a labor market that is oriented to the service sector leads citrus growers towards a separation of professionals. That is, full-time growers and non-professional part-time growers will retain their orchards for traditional or sentimental reasons.

Drip irrigation is the main irrigation system for citrus in Spain, and it is used in almost 84% of the citrus cropped area [2]. The modernization of irrigation infrastructures carried out by the central and regional governments of Spain in the last two decades improved drip irrigation. This led to a change from old channel networks and surface irrigation systems to pressurized irrigation networks and drip irrigation systems at the orchard level [3,4,5].

The importance of irrigation systems to secure food for a growing population initiated the international research program on irrigation performance, which is currently the International Water Management Institute of CGIR. Irrigation performance assessment was performed based on the adequacy, efficiency, dependability, and equity of irrigation water delivery [6,7]. Further developments [8,9,10,11] established the fundamentals of performance assessment using benchmarking and performance indicators and extended the performance criteria to productivity, profitability, and sustainability criteria [11,12,13].

Irrigation performance assessment examples can be found in Spain for irrigation schemes in Andalusia [14,15,16], Murcia Region [17,18], Aragon [19], and Valencian Community [20,21]. Some examples of irrigation performance assessment around the world include research works carried out in Turkey [22,23], Uzbekistan [24], Iran [25], Ethiopia [26,27], Rwanda [28], Sudan [29], Tanzania [30], China [31], India [32,33,34], Sri Lanka [35], South Korea [36], Brazil [37], and Mexico [38], among others.

Classification of irrigation schemes using benchmarking techniques has attracted the attention of fewer authors. The application of data envelopment analysis (DEA) was proposed as a methodology to overcome the problems related to the lack of ways to assign the correct weightings for the calculation of indexes and to the subjectivity of the interpretation of comparative results [39,40,41]. The principal components approach has also been used as a means to quantify and rank the performance of selected irrigation schemes in Kenya [42] and Turkey [43]. Zema et al. [44] introduced the use of permutational multivariate analysis of variance (PERMANOVA), multidimensional scale models (MDS), and distance-based linear Models (DISTLM) to evaluate the technical and financial performances of water user associations in Southern Italy. A linear combination of adequacy, efficiency, equity, and dependability indicators was employed in Reference [45] to measure and compare the overall performance of an irrigation scheme over time. To analyze the impact of water pricing policies, a comparative scorecard assessment approach was developed in Jordan and Iran [46] using positive mathematical programming (PMP) [47]. A two-stage fuzzy differential water price model was applied for water rights allocation in China [48].

The analytic hierarchy process [49] was recently applied to the evaluation of irrigation and drainage networks based on virtual water and water productivity instead of the performance indicators used normally.

Several authors have introduced the use of AHP in the field of agricultural research. Alphonce [50] suggested the potential application of AHP in agricultural decision-making such as resource allocation, subsistence farming, or determination of the production technology. Other studies focus on the development of decision-making models; to select the most appropriate irrigation method [51,52], to select irrigation water optimal allocation [53,54], for the assessment of irrigation water quality [55], for the evaluation of agricultural water management [56], and in the improvement of irrigation projects [57]. However, no AHP studies have been found aimed at the construction of an integrative and comprehensive indicator of irrigation efficiency.

To help farmers assess the irrigation efficiency of their orchards, a new multicriteria approach based on the analytic hierarchy process (AHP) technique and the participation of a group of experts is proposed.

In this work, we propose to apply the analytic hierarchy process (AHP) technique in the development of a comprehensive index and then use the resulting index in the classification of orange growers orchards’ in an irrigators community in Valencia (Spain). This study is in the framework of the project 2019ES06RDEI7346 Improving the use of water and energy in modernized irrigation of fruit trees (GO InnoWater) funded by the Spanish Rural Development Program (2014–2020). This project has the main objective of developing useful tools to help irrigators communities (IC) in the irrigation decision-making process.

We propose a methodology that identifies performance irrigation indicators with the participation of experts. The methodology is based on the AHP approach and allows the aggregation of expert judgments on each of the selected indicators used into one irrigation efficiency index (IEI). This methodology develops a synthetic index of water irrigation efficiency based on the relative weights of selected indicators using the AHP technique.

## 2. Material and Methods

The AHP-based approach has been proposed in this work to develop a benchmarking tool to assess irrigation water use efficiency.

The AHP method is briefly described in Section 2.1, the proposed methodology, and its application to a case study in Section 2.2.

### 2.1. The Analytic Hierarchy Process Overview

The AHP technique formalizes and performs a systematic decision-making process that is largely subjective and, therefore, facilitates “accurate” judgments. As a result of the method, decision-makers receive information on the implicit weights of the evaluation criteria. The AHP method comprises the following steps:

1. Identification of the problem: Before starting any numerical calculation, it must be verified that the problem in question can be presented as a structured model, where the criteria and the alternatives of the process are identified.
2. Selection of criteria: In this stage, the criteria associated with the multi-criteria decision-making process are selected, which will be assessed and weighted in later stages.
3. Pairwise comparison of criteria: Each criterion *i* is compared with criterion *j* using the relative priority Saaty’s 1–9 scale [58].
4. Priority calculation: The weights of each one of the criteria are calculated.
5. Coefficient of consistency calculation: This coefficient measures the degree of homogeneity between the judgments issued by the experts or stakeholders of the process. A value less than 0.1 is considered admissible.

A detailed description of these steps and the mathematical formulation of the AHP method can be found in Reference [59].

### 2.2. Proposed Methodology: An AHP Modeling Approach

The main objective of this work is to propose a methodology based on the analytic hierarchy process (AHP) technique and the participation of a group of experts in the area of agricultural productivity and economics to assess irrigation water use efficiency. This methodology was applied in a case study to analyze the results obtained.

The criteria used in this methodology were the key performance indicators used as a benchmarking tool among orchards to assist farmers in their decision-making process.

**Figure 1:**
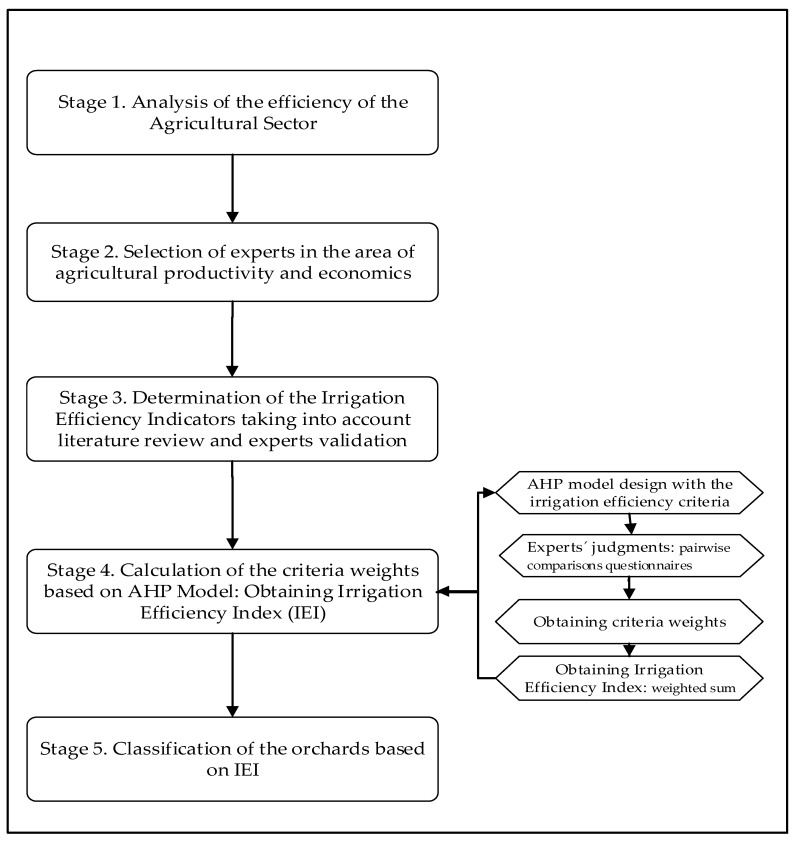
Proposed methodology.

Figure 1 shows the five stages of the methodology proposed in this study.

This methodology was applied to 24 orange orchards in the Valencian community during the 2017 cropping season. All of the orchards were of adult navelina variety trees, and all of them belonged to the same irrigator community and the same farmer’s cooperative.

The irrigator community shared data about the annual water consumption of each orchard and the cooperative conveyed information about the fruit grade and yield, according to the harvest records of each farmer. Both associations allowed the use of shared information for research purposes but while retaining the confidentiality of the information. Among all the included crops in the database, this work analyses the navelina variety because it is the most widely cultivated, with the largest number of orchards.

These orchards were very close to each other, and had very similar soil and other physical characteristics, such as the age of the trees, planting density, or cultivation practices.

The sizes of the orchards and the type of farmers (full-time and part-time) were representative of the Valencian citriculture with sizes between 0.3 and 1.7 ha and around half of the farmers being part-time [60]. The effect of the type of farmer, i.e., full time or part-time, and the size of the orchard were evaluated based on the irrigation efficiency of the analyzed orchards because these factors are related to two of the main problems in Valencian agriculture productivity: the small size of farms and part-time farmers [60,61].

Stage 1: Analysis of the efficiency in the agricultural sector. 

The analysis of the productive efficiency in the area of agricultural productivity and economics allows us to define the factors that more strongly affect irrigation efficiency. These factors are used to lay the foundations for the irrigation productive performance of the agricultural sector.

The model aims to obtain an IEI for the orchards in the case study to compare their relative position to the productive performance of the agricultural sector. The model will allow us to classify the orchards depending on the index obtained. For the irrigation efficiency measurement among orchards, it is necessary to select orchards belonging to the same geographical area.

Stage 2: Definition of the experts in the area of agricultural productivity and economics.

Following [62], this methodology considers the opinion of several experts to provide a more objective prioritization.

When the information available is uncertain, it is necessary to make estimates. In such cases, experience and knowledge of the problem are more important than the prioritization technique itself. Therefore, it is preferable to focus the efforts on finding a group of proficient experts and getting them involved in the process.

Three experts were selected for their experience and in-depth knowledge about the problem.

Expert 1: Holds a Ph.D. in Agricultural Engineering from the Castilla-La Mancha University. He is the Dean of the Higher Polytechnic School of Orihuela, and a professor in the field of hydraulics and irrigation at the Engineering Department of Miguel Hernández University of Elche (Alicante). He is a specialist in pressurized irrigation networks and has more than 25 years of experience in research, teaching, and innovation in the area of hydraulics and irrigation.

Expert 2: Holds a Ph.D. in Agricultural Engineering from the Miguel Hernández University of Elche. He is a founding partner of MOVAL Agroingeniería, an engineering services company specializing in irrigation, management of irrigation communities, energy optimization, construction projects, and works direction, among others. He has more than 10 years of experience in the area of hydraulics and irrigation engineering.

Expert 3: Holds an agricultural engineer and an industrial technical engineer qualification from the Polytechnic University of Valencia. He has more than 25 years of experience in managing irrigation communities as an external advisor for fertigation and energy efficiency programs in citrus and fruit tree crops. He also has broad experience in designing projects and works direction in the area of irrigation facilities modernization.

Stage 3: Determination of the irrigation efficiency indicators (IEIs) considering the literature review and expert validation.

The three experts in the area of agricultural irrigation management selected in stage 2 of the methodology participated in this stage. After the analysis of the literature, the most relevant criteria were presented to these experts in a participatory workshop. The group of experts determined which factors were more important in irrigation efficiency and defined the four indicators for irrigation efficiency measurement by consensus. These indicators were the AHP model criteria.

These IEIs were defined in a participatory workshop within the framework of the *GO InnoWater* project through discussion and subsequent agreement between the experts in irrigation management and agricultural economics from the project partners.

These four indicators are:

1. Annual relative irrigation supply, RIS (%), which measures the service delivery performance
  $$\mathrm{RIS}=\frac{\mathrm{Total}\;\mathrm{annual}\;\mathrm{volume}\;\mathrm{of}\;\mathrm{irrigation}\;\mathrm{water}\;\mathrm{applied}}{\mathrm{Total}\;\mathrm{annual}\;\mathrm{volume}\;\mathrm{of}\;\mathrm{crop}\;\mathrm{irrigation}\;\mathrm{demand}}=\frac{I}{{\mathrm{ET}}_{c}-P_{e}}$$
  where:
  P_e_ is the annual effective precipitation or rainfall.
  I is the annual irrigation water applied to the crop.
  ET_c_ is the annual crop evapotranspiration calculated according to FAO-56 [63].

The optimal RIS value is 100%, that is when irrigation applied matches the water requirement of the crop. This maximization criterion implies that the RIS should be transformed into [100-Abs (100-RIS)] in case there were orchards where the irrigation supply exceeded the calculated optimum (100%).

2. Output per unit irrigation delivery, OUI (€/m^3^). This indicator measures the financial efficiency:
  $$\mathrm{OUI}=\frac{\mathrm{Total}\;\mathrm{annual}\;\mathrm{value}\;\mathrm{of}\;\mathrm{crop}\;\mathrm{production}}{\mathrm{Total}\;\mathrm{annual}\;\mathrm{volume}\;\mathrm{of}\;\mathrm{irrigation}\;\mathrm{water}\;\mathrm{applied}\;\mathrm{to}\;\mathrm{the}\;\mathrm{crop}}$$
3. Overall efficiency of the irrigated crop. A physical irrigation water productivity, WUE_overall_, was an indicator that was included to account for year-to-year price variation that could be remarkable for certain years. WUE_overall_ is defined as the ratio between the total annual mass of crop production, including all grades, and the annual volume of irrigation water applied (kg/m^3^).
  $${\mathrm{WUE}}_{\mathrm{Overall}}=\frac{\mathrm{Total}\;\mathrm{annual}\;\mathrm{mass}\;\mathrm{of}\;\mathrm{crop}\;\mathrm{production}}{\mathrm{Total}\;\mathrm{annual}\;\mathrm{volume}\;\mathrm{of}\;\mathrm{irrigation}\;\mathrm{water}\;\mathrm{applied}\;\mathrm{to}\;\mathrm{the}\;\mathrm{crop}}$$
4. The efficiency of the irrigated crop for a first-class yield: As product quality is a key performance indicator, and highly related to irrigation in Mediterranean fruticulture, WUE_Quality_ is defined here as the ratio of the first-class yield measured from the field at harvest and the total volume of irrigation water applied to the crop (kg/m^3^). This latter indicator is equivalent to the water use efficiency indicator defined by Reference [64].
  $${\mathrm{WUE}}_{\mathrm{Quality}}=\frac{\mathrm{First}\;\mathrm{class}\;\mathrm{annual}\;\mathrm{mass}\;\mathrm{of}\;\mathrm{crop}\;\mathrm{production}}{\mathrm{Total}\;\mathrm{annual}\;\mathrm{volume}\;\mathrm{of}\;\mathrm{irrigation}\;\mathrm{water}\;\mathrm{applied}\;\mathrm{to}\;\mathrm{the}\;\mathrm{crop}}$$

Stage 4: Calculation of the criteria weights based on AHP Model: Obtaining IEI

This step aims to obtain an index for each orchard, which indicates the level of irrigation productive performance according to all the indicators considered, the IEI. The higher the value of the index, the more efficient the orchard in using irrigation water.

This stage covers the following steps.

4.1: AHP model design with the irrigation efficiency indicators. Four indicators were built up.

4.2: Expert judgments: pairwise comparisons questionnaires. With the help of the three experts, the importance of each criterion of the model on the others (indicators) was determined using Saaty’s scale [59] for the pairwise comparisons through questionnaires specifically designed for that purpose (see Appendix A).

4.3: Obtaining criteria weights. The individual expert judgments were processed using the © 2021–2020 Expert Choice Software (v.6.0.013.38933, Expert Choice Inc., Arlington, VA, USA) for the priority calculations described in Section 2.1. The aggregation of the individual judgments was carried out using the geometric mean of the priority vectors of the criteria of each expert [59]. The value indicates the importance of the irrigation efficiency indicators in the evaluation model.

4.4: Obtaining IEI: the weighted sum. With this method, a dimensionless value was obtained for each orchard assessed. The value indicates the relative position of the orchard compared to that of the other orchard, the IEI.

The consistency of the decision-making process was also calculated in this step. These results are shown in Table 1.

According to the results presented in Table 1, we can see that there was a priority criterion among the judgments made by the experts, who prefer the output per unit irrigation delivery as a predominant criterion for measuring irrigation efficiency. However, the four most relevant criteria were considered for irrigation efficiency measurement since they were selected as common measurement indicators in agricultural productivity and were accepted by consensus among all the experts.

As can be seen in Table 1, the judgments issued by the experts were consistent since the consistency coefficient (CC) was less than 0.1. As highlighted before, the most important criterion was the output per unit irrigation delivery with 41.08%. This was because this criterion measures the income from a crop in relation to the volume of irrigation, i.e., the efficiency of a crop in financial terms. The overall efficiency of the irrigated crop criterion with 27.79% occupied the second place since it measures the efficiency of a crop in quantitative terms of production.

Stage 5. Multi-criteria orchards classification based on IEI.

Finally, in this stage, the orchards were ordered based on the level of compliance of each orchard for the four criteria, thereby obtaining the results in Table 2 in Section 3. The results of the classification of the orchards according to the irrigation efficiency indicators led to obtaining the IEI for each orchard. This IEI was calculated using the weighted sum of the standardized values of each orchard for each criterion (Table 3). To aggregate all the four values of the indicators for each orchard, these indicators must be the criteria to be maximized.

## 3. Results

The OUI, WUEoverall, WUEQuality criteria were the criteria that should be maximized. The RIS value is calculated with respect to the optimal RIS value (100%) when irrigation matches the water requirement of the crop. In this study, no orchard exceeded the maximum water requirements of these crops (i.e., there was no RIS value higher than 100%), the RIS indicator was also a criterion to be maximized in this study, without the need of being transformed in the way shown before when describing the RIS indicator calculation.

The values for each of the four criterions were obtained for the 24 orchards of adult navelina belonging to the same irrigation community (IC) and cooperative of farmers. Table 2 shows these results.

As observed in Table 2, there were two outliers, orchard 7 with a RIS value of 10.40% and orchard 15 with a RIS value of 92.18%. These two orchards were reviewed. Orchard 7 was found abandoned after 2017 (the reason that explains the extremely low value of the irrigation applied), and orchard 15 had a technical fault in the irrigation system programmer in 2017 that led to excessive and uncontrolled irrigation.

After removing these two outliers, the distribution of the RIS values in a range between 16% and 72% showed that the total annual volume of irrigation water applied to the crop was well below the calculated crop irrigation requirements, and this could be explained by water scarcity in the Mediterranean area [65]. Due to this scarcity, the IC charges a high extra price for the amount of water exceeding the RIS value of 70%, which prevents the consumption of irrigation water above this value.

To classify the orchards according to their IEI, the values of each orchard for each indicator were normalized and multiplied by their corresponding weight resulting from the AHP method. The normalization allowed comparisons independent of the unit of measurement (measurement scale) and was carried out following the standardization method (z-score). It was calculated by subtracting the mean of the orchards values for each indicator from each value and then dividing these differences by the standard deviation (SD) of the orchards values obtained for each indicator. Later, the orchards were ordered in descending order. Table 3 shows the results of this stage. Additionally, the type of farmer and size of each orchard were included in these results to characterize the most efficient orchards.

On one hand, the results of Table 3 seem to refute the initial hypothesis that irrigation water use efficiency increases with the size of the orchards. IEI standardized (IEIz) did not show any linear correlation with the size of the orchard (determination coefficient R2 equal to 0.01, calculated as the square of the Pearson correlation coefficient between the two variables).

On the other hand, the results of Table 3 seem to be in accordance with the initial hypothesis that full-time farmers use irrigation water in a more efficient way than part-time ones. As highlighted in Table 3, four out five orchards with the highest IEIz values belong to full-time farmers (highlighted in the bold letter within the table) and four out five orchards with the lowest IEIz values belong to part-time farmers (highlighted in the italic letter within the table). Besides, full-time farmers also obtain higher values in all four indicators, as can be observed in Table 3 and Table 4.

Table 4 also shows an average positive value of 0.27 for the IEIz among full-time farmers and a negative value of −0.2225 for the IEIz among part-time farmers.

High variability detected in all water irrigation efficiency indicators of Table 2 was discussed in a participatory workshop held in 2020 July with farmers from the *Go InnoWater* project. The conclusions of this participatory workshop in which farmers were asked: “How do we irrigate fruit trees?” can be summarized in the following list:

- According to actual weather (rain, temperature, humidity, wind…).
- According to the forecast weather.
- According to the type of crop.
- Own criteria (timing according to personal experience).
- Starting with 2 h/day and increasing until harvest.
- By type of land (observing the soil moisture).
- By the availability of water and price.
- By the age of the crop and expected production.
- By the expected price of the product.
- According to the recommendations of IVIA (Valencian Institute of Agrarian Research) [66].
- Observing the plant appearance.
- Two patterns: before or after harvest.

The participant farmers in the workshop also proposed the actions to be taken by the project to improve the irrigation management in the IC. The most important proposal was training and assistance for farmers around plant water needs knowledge and new technologies for irrigation management. This training and assistance should lead to a homogenization of the irrigation indicators based on the identification of the best irrigation practices among the farmers of the IC with the help of the benchmarking tool presented in this article.

## 4. Conclusions

The present paper describes a new approach, based on the AHP technique, to assess irrigation water use efficiency reliably at the plot or orchard level. First, it includes an indicators selection process adapted to the particular case of irrigated citrus trees in the Mediterranean area of Spain. Second, we have developed a standardized synthetic index of irrigation water use efficiency (IEIz) based on the relative weights of the four selected indicators that have been ranked according to the experts’ opinions using AHP. Finally, this synthetic index is tested with real data to show its utility as a benchmarking tool for orchards of the same or similar crop to assist farmers in their decision-making processes.

The use of AHP is justified by its ability to obtain quantitative values from expert qualitative judgments and because it enables the aggregation of the selected expert assessments. The three experts were chosen according to their experience and knowledge in the area of agricultural productivity and economics. The AHP method allowed better communication, leading to a clearer understanding and consensus in the indicators’ selection among these three experts.

The weighting of the indicators provides some important insights into the general conception of the experts on irrigation efficiency. The data resulting from the indicators show that the most important criterion is output per unit irrigation delivery (OUI in €/m^3^) as a predominant criterion for measuring irrigation efficiency (weight of 41.08%), reflecting that agriculture is an economic activity in which income is more important than yield amount and quality of the fruit. Besides, the low weight of the RIS criterion (15.44%) could be explained by the use of deficit irrigation scheduling in Mediterranean citriculture.

This synthetic index has been tested in one IC of Valencia, using data from 24 orchards of adult navelina citrus variety with very similar agronomical characteristics and representative of the Valencian citriculture. The IEI allows us to compare their relative position of an orchard to the productive performance of the agricultural sector.

The results obtained in this test have shown a high range of variability in the four original indicators (Table 2), which reinforce the importance of evaluating the efficiency in the use of irrigation water as over 70 percent of freshwater in Spain is used for agriculture [1]. The reasons for this high variability have been analyzed considering the size of the orchard and the type of farmer (full-time or part-time farmer). The size of the orchards did not show any correlation with the IEIs but full-time farmers obtain better scores in the mean value of every indicator.

This methodology can be replicated in different IC as an internal benchmarking tool to assess irrigation water use efficiency and to identify the best irrigation and agricultural practices among the farmers.

Finally, we found some obstacles to the spread of the methodology presented in this study not directly related to the technical procedure nor its theoretical basis. These obstacles are mainly related to the problems with trust in private farm data sharing in Europe exposed by Reference [67]. How to overcome these sharing problems, the problems related to the interoperability of data from different sources, and the development of software packages to benchmark the efficiency of irrigation water use are the three main research areas that will require attention in the near future.

## Figures and Tables

**Table 1:** Application of stage 4 of the methodology. AHP method results.

Calculation of Criteria Weights and Consistency Index (Step 4)	RIS: 15.44% OUI: 41.08% ${\mathrm{WUE}}_{\mathrm{Overall}}$: 27.79% ${\mathrm{WUE}}_{\mathrm{Quality}}$: 15.69% C.I: 0.0021 R.I: 0.9 C.C: 0.0024

**Table 2:** Values of the four indicators for the 24 orchards. Outliers included (*).

Orchard	Annual Relative Irrigation Supply RIS (%)	Output Per Unit Irrigation Delivery. OUI (€/m^3^)	Overall Efficiency of the Irrigated Crop. WUE_overall_ (kg/m^3^)	Efficiency of the Irrigated Crop for First Class Yield. WUE_Quality_ (kg/m^3^)
1	49.32%	4.48 €	14.02	9.66
2	30.59%	3.51 €	23.06	9.11
3	33.71%	2.93 €	12.87	11.95
4	71.81%	1.50 €	6.97	5.54
5	37.49%	2.57 €	18.75	5.53
6	34.20%	2.55 €	23.41	2.71
(*) 7	10.40%	7.34 €	35.64	27.36
8	48.21%	1.41 €	10.30	3.04
9	45.36%	1.37 €	6.08	5.51
10	21.86%	2.50 €	18.25	5.53
11	57.79%	0.99 €	8.68	0.89
12	16.41%	2.25 €	19.76	2.03
13	26.05%	1.49 €	15.31	0.55
14	39.62%	1.05 €	6.27	3.16
(*) 15	92.18%	0.77 €	5.28	1.94
16	53.49%	0.83 €	4.70	2.61
17	32.44%	1.12 €	10.44	0.55
18	16.84%	1.88 €	18.62	1.18
19	54.78%	0.68 €	4.47	1.80
20	16.08%	1.24 €	7.04	3.91
21	36.80%	0.61 €	5.22	0.91
22	20.49%	0.83 €	7.67	0.87
23	23.14%	0.73 €	6.90	0.64
24	23.96%	0.57 €	2.95	1.97

**Table 3:** Twenty-two remaining orchards ordered according to their IEI standardized.

Orchard	RIS Z-Score (%)	OUI Z-Score (€/m^3^)	WUE_Overall_ Z-Score (kg/m^3^)	WUE_Quality_ Z-Score (kg/m^3^)	Irrigation Efficiency Index Z-Score (IEIz)	Type of Farmer (Full-Time, Part-Time)	Size of the Orchard (Ha)
1	0.877	2.687	0.401	1.876	1.65	FT	0.497
2	−0.350	1.751	1.808	1.706	1.44	PT	0.3039
3	−0.145	1.196	0.222	2.587	0.94	FT	0.9815
6	−0.113	0.835	1.862	−0.284	0.80	FT	1.745
5	0.102	0.854	1.138	0.593	0.78	FT	0.5668
10	−0.922	0.783	1.060	0.594	0.57	PT	0.5314
12	−1.279	0.547	1.294	−0.494	0.31	PT	0.6826
4	2.351	−0.175	−0.697	0.596	0.19	FT	0.514
18	−1.250	0.189	1.117	−0.758	0.08	PT	0.2822
8	0.804	−0.263	−0.178	−0.182	−0.06	FT	0.6568
13	−0.647	−0.192	0.601	−0.954	−0.16	FT	0.7179
9	0.618	−0.309	−0.834	0.586	−0.17	FT	1.708
11	1.432	−0.670	−0.430	−0.848	−0.31	PT	0.2705
17	−0.229	−0.546	−0.156	−0.954	−0.45	FT	1.1538
14	0.242	−0.609	−0.805	−0.142	−0.46	PT	1.1022
16	1.150	−0.827	−1.050	−0.314	−0.50	PT	0.668
20	−1.300	−0.427	−0.686	0.090	−0.55	PT	0.3792
19	1.235	−0.971	−1.085	−0.565	−0.60	PT	0.5552
22	−1.012	−0.821	−0.587	−0.854	−0.79	PT	0.6826
21	0.057	−1.032	−0.969	−0.842	−0.82	FT	1.7056
23	−0.838	−0.922	−0.706	−0.927	−0.85	PT	0.5241
24	−0.784	−1.077	−1.322	−0.513	−1.01	PT	0.4734
Mean	0.000	0.000	0.000	0.000	0.000		
SD	1.000	1.000	1.000	1.000	0.752		

**Table 4:** Cross-tabulation of the mean value of the IEI standardized, by type of farmer.

Variable	Indicators	Total Sample	Type of Farmer	
			Full Time Famers	Part Time Farmers
IEI Z-Score	Mean Number of orchards	0.0014 *n* = 22	0.2700 *n* = 10	−0.2225 *n* = 12
RIS Z-Score	Mean Number of orchards	0.0000 *n* = 22	0.3675 *n* = 10	−0.3063 *n* = 12
OUI Z-Score	Mean Number of orchards	0.0000 *n* = 22	0.3055 *n* = 10	−0.2545 *n* = 12
WUE_overall_ Z-Score	Mean Number of orchards	−0.0001 *n* = 22	0.1390 *n* = 10	−0.1160 *n* = 12
WUE_Quality_ Z-Score	Mean Number of orchards	−0.0001 *n* = 22	0.3022 *n* = 10	−0.2521 *n* = 12

## Appendix A

### Questionnaire AHP Criteria Weighting

For each pair of criteria please indicate highlighting in black which of the two you consider to be most important and to what extent. Remember that these are criteria to be used in the assessment of irrigation water use efficiency.

The criteria must be compared pairwise, asking to what degree criterion Ci is better compared with criterion Cj, using the following scale (Saaty’s scale):

**Cij = 1: considered equally important criterion i and criterion j**
**Cij = 3: criterion i is considered slightly more important than criterion j**
**Cij = 5: criterion i is considered considerably more important than criterion j**
**Cij = 7: criterion i is considered much more important (or demonstrably more important) than criterion j**
**Cij = 9: criterion i is considered absolutely more important than criterion j**

**------------------------------------------------------------------------------------------------------------------------**

**C1: Annual Relative Irrigation Supply, RIS (%)**
**C2: Output per unit irrigation delivery, OUI (€/m^3^)**

Which objective do you consider more important?	C1	C2			
To what extent?	1	3	5	7	9

**C1: Annual Relative Irrigation Supply, RIS (%)**
**C3: Overall Efficiency of the irrigated crop**

Which objective do you consider more important?	C1	C3			
To what extent?	1	3	5	7	9

**C1: Annual Relative Irrigation Supply, RIS (%)**
**C4: Efficiency of the irrigated crop for first class yield**

Which objective do you consider more important?	C1	C4			
To what extent?	1	3	5	7	9

**C2: Output per unit irrigation delivery, OUI (€/m^3^)**
**C3: Overall Efficiency of the irrigated crop**

Which objective do you consider more important?	C2	C3			
To what extent?	1	3	5	7	9

**C2: Output per unit irrigation delivery, OUI (€/m^3^)**
**C4: Efficiency of the irrigated crop for first class yield**

Which objective do you consider more important?	C2	C4			
To what extent?	1	3	5	7	9

**C3: Overall Efficiency of the irrigated crop**
**C4: Efficiency of the irrigated crop for first class yield**

Which objective do you consider more important?	C3	C4			
To what extent?	1	3	5	7	9
